# *CRB1-*Related Leber Congenital Amaurosis: Reporting Novel Pathogenic Variants and a Brief Review on Mutations Spectrum

**DOI:** 10.29252/.23.5.362

**Published:** 2019-09

**Authors:** Mohammad Saberi, Zahra Golchehre, Arezou Karamzade, Mona Entezam, Yeganeh Eshaghkhani, Elaheh Alavinejad, Hassan Khojasteh Jafari, Mohammad Keramatipour

**Affiliations:** 1 Department of Medical Genetics, School of Medicine, Tehran University of Medical Sciences, Tehran, Iran;; 2 Department of Medical Genetics, School of Medicine, Shiraz University of Medical Sciences, Shiraz, Iran;; 3 Farabi Eye Hospital, Tehran, Iran

**Keywords:** CRB1, Leber congenital amaurosis, Retinal dystrophies, Whole exome sequencing

## Abstract

**Background:**

Leber congenital amaurosis (LCA) is a rare inherited retinal disease causing severe visual impairment in infancy. It has been reported that 9-15% of LCA cases have mutations in *CRB1* gene. The complex of CRB1 protein with other associated proteins affects the determination of cell polarity, orientation, and morphogenesis of photoreceptors. Here, we report three novel pathogenic variants in *CRB1* gene and then briefly review the types, prevalence, and correlation of reported mutations in *CRB1 *gene.

**Methods:**

Whole exome sequencing and targeted gene panel were employed. Then validation in the patient and segregation analysis in affected and unaffected members was performed.

**Results:**

Our detected novel pathogenic variants (p.Glu703*, c.2128+1G>A and p.Ser758SerfsX33) in *CRB1* gene were validated by Sanger sequencing. Segregation analysis confirmed the inheritance pattern of the pathogenic variants.

**Conclusion:**

Our findings show that emerging the next-generation sequencing-based techniques is very efficient in identifying causative variants in disorders with locus heterogeneity.

## INTRODUCTION

Leber congenital amaurosis (LCA) was first described by Theodore Leber in 1869. It is the most severe form of all inherited retinal diseases^[^^1^^,^^2^^]^. LCA is a group of inherited retinal dystrophies characterized mainly by severe visual impairment, nystagmus, and severely subnormal or non-detectable electroretinogram (ERG)^[^^2^^-^^6^^]^. Other common clinical features of the disorder are Franceschetti's oculo-digital sign (a pathognomonic sign of LCA), photophobia, hyperopia, and keratoconus^[^^5^^,^^6^^]^. Incidence of LCA is 2-3 per 100,000 newborns although it is more prevalent in isolated populations and in populations with high consanguinity rates^[^^1^^,^^5^^]^.

LCA is a genetically heterogeneous disease that, in most cases, is inherited in an autosomal recessive manner^[^^2^^,^^4^^,^^5^^,^^7^^]^. However, there are few autosomal dominant mutations, notably in *CRX* gene. Nearly, 30 genes have been identified to cause LCA, which* CEP290*, *GUCY2D*, and *CRB1* are the most frequently mutated genes^[^^5^^,^^8^^-^^10^^]^. Identified genes explain approximately 70% of molecular basis of the disease; however, the remaining 30% are unresolved cases^[^^5^^,^^6^^]^.

**Fig. 1:**
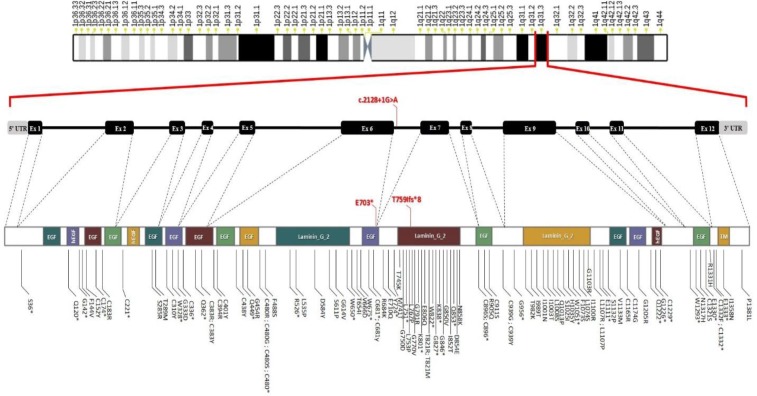
Spectrum of mutations in functional domains of CRB1. Our novel pathogenic variants are illustrated with red color. The schematic representation of CRB1 domains was obtained from Phosphosite database (https://www.phosphosite.org/proteinAction?id= 11964200&showAllSites=true). EFG, EGF-like domain (Pfam: PF00008); hEGF, human growth factor-like EGF (Pfam: PF12661); Laminin_G_2, laminin G domain (Pfam: PF02210); TM, transmembrane

*Crumbs (Drosophila) homolog 1* (*CRB1*) gene encodes a transmembrane protein expressed in brain and retina. Protein CRB1 plays a role in determining and maintaining the apical polarity and adherent junction in embryonic epithelia^[^^11^^]^. For the first time, the causative effect of *CRB1* gene on LCA disease has been shown by Lotery et al.^[^^12^^]^. Their cohort study has revealed that *CRB1* accounts for 9% of LCA cases. Other studies have reported its attribution to LCA patients ranged from 9% to 15%^[^^6^^,^^11^^,^^13^^]^. Based on a literature review, the spectrum of reported mutations in functional domains of *CRB1 *gene can cause LCA (Fig. 1).

Application of next-generation sequencing (NGS) has been very advantageous for detecting causative genetic variants in monogenic diseases, especially disorders with locus heterogeneity^[^^14^^,^^15^^]^. Accordingly, this technology has been employed to detect the molecular causes of retinal dystrophies in many studies and showed impressive detection rates^[^^16^^-^^19^^]^.

In this study, we report three novel pathogenic variants in three Iranian families with LCA, which has been detected by whole exome sequencing (WES) and targeted gene panel.

## MATERIALS AND METHODS

**Subjects**

**Fig. 2:**
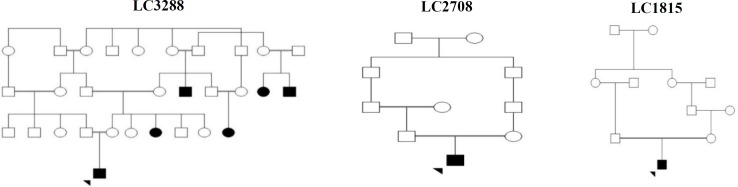
Pedigrees of the investigated families. Probands were indicated by arrowheads

Here, we report three pedigrees of our cohort for 117 patients with inherited retinal diseases, which will be published in near future. Primary clinical diagnosis was established by an expert ophthalmologists through medical history, clinical vision evaluations such as funduscopy, family history, and paraclinical investigations such as ERG. Available relatives (affected or unaffected) were invited to submit a blood sample for segregation analysis. The pedigrees of the investigated cases are illustrated in Figure 2. An informed consent was obtained from all the participants or their parents/guardians. This study was approved (IR.TUMS.MEDICINE.REC.1396.4198) by the Research Ethics Committee of Tehran University of Medical Sciences, Tehran, Iran.

Genomic DNA was extracted from whole blood using Exgene™ Blood SV DNA purification kit (GeneAll^®^, Korea) according to the manufacturer’s instructions. Considering great genetic heterogeneity of the suggested diagnosis, cone-rod dystrophy (CRD), NGS-based methods were used for the probands. WES was done for proband of LC3288 pedigree, and targeted panel including 19 genes were performed for LC1815 and LC2708 pedigrees. SureSelect V6™ Target Enrichment Kit (Agilent, USA) with the 61-Mb target region was used in WES and NimbleGen, Roch™ comprised 19 genes (69,628 base pairs) for targeted panel. The enriched libraries were sequenced on the Illumina Hiseq 4000™ platform. Sequencing reads were generated in the FASTQ format after nucleotide calling. Read pairs were aligned to the human reference genome (hg19) using the Burrows-Wheeler Aligner, and duplicate reads were marked with PICARD tools. The GATK Unified Genotyper module was applied for Indel realignment, base recalibration, variant calling, and variant filtering. Then variants were annotated using SnpEff tool. Variant prioritization based on population frequency, effect or nature of the variants, and zygosity were performed.

**Genetics Analysis**

Polymerase chain reaction (PCR) followed by Sanger sequencing was conducted to confirm the variants of interest in the probands. Besides, the investigations of the detected variants in their available members of the pedigrees were carried out by PCR-Sanger sequencing. The PCR products were purified by Expin^TM^ Combo GPMini purification kit (GeneAll Biotechnology, Seoul, South Korea) and sequenced by ABI 3500 automated sequencer (Pishgam Biotech Company, Tehran, Iran).

**Variant** **interpretation**

Allelic frequency of the variants was investigated in population databases viz dbSNP (http://www.ncbi.nlm. nih.gov/snp), 1000 genome project (1000GP) (http://browser.1000genomes.org), Exome Aggregation Consortium (ExAC) (http://exac.broadinstitute.org/), NHLBI GO Exome Sequencing Project (ESP) (http://evs.gs.washington. edu/EVS), and our local database that includes more than 1000 exome of Iranian population. A variety of computational (*in silico*) predictive tools, including MutationTaster (http://www. mutationtaster. org)^[^^20^^]^, Combined Annotation-Dependent Depletion (CADD; http://cadd. gs.washington.edu)^[^^21^^]^, and DANN Score^[^^22^^]^ were used to evaluate the effect of the detected nucleotide exchange on the basis of evolutionary conservation, protein structure, and protein function. Moreover, the variant was investigated in RetNet (https://sph.uth.edu/ retnet/), HGMD (Human Gene Mutation Database), ClinVar (https://www.ncbi. nlm.nih.gov/clinvar), GeneReviews^®^ (https://www. ncbi.nlm.nih.gov/books/ NBK1116/), and OMIM^®^ databases and also in literature for any previously reported record.

## RESULTS

**Clinical findings**

Based on clinical and para-clinical examinations, LCA was suggested as primary clinical diagnosis of patients. Moreover, there was no evidence for the involvement of other organs; no signs of intellectual disability, skeletal abnormality, hearing impairment, and renal malfunction were observed.

**Genetic** **findings**

**Fig. 3:**
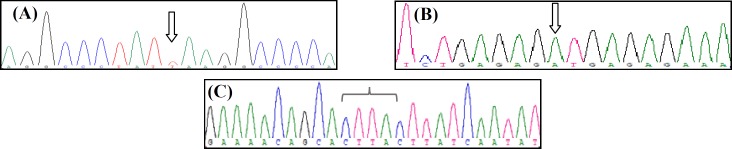
Genotypes of the probands. (A) c.2107G>T in LC3288; (B) c.2276_2279dupCTTA in LC2708; (C) c.2128+1G>A in LC1815. Arrows show position of the variants

Performed WES generated approximately 161 million reads, with 137 million non-redundant reads mapped to the target regions. More than 99% of the target regions had >10coverage, and more than 96% of the target regions had >30 coverage. In average, performed targeted panel covered 98.8% of target regions with an average depth of 250. Moreover, 93.5% of the target regions were covered by 30. Step by step analysis and filtering of the NGS data left three homozygous variants in *CRB1* gene (**Table 1**). The variants were absent from population databases (ExAC, 1000G, dbSNP, and our local database) and have not previously been reported in the literature. In addition, as shown in **Table 1**, multiple lines of *in silico* computational analysis support the deleterious effect of the variants on the gene or gene product (MutationTaster, CADD, and DANN scores). Homozygosity of the probands was also confirmed by PCR-Sanger sequencing (Fig. 3). 

## DISCUSSION

LCA, as the most severe and early onset form of inherited retinal diseases, is responsible for approximately 20% of blind children studying in schools^[^^1^^,^^2^^,^^4^^]^. Genetic testing is a crucial step for patients suspected to have LCA because it has the ability to confirm diagnosis and makes distinction between LCA and other retinal diseases with similar clinical presentations. Determining the causative variant is very helpful in genetic counselling and future reproductive planning for the family. It may also help in defining the prognosis of the disease, as well as making personalized decision for the patient, including mutation or gene-specific treatments in near future.

**Table 3:** Description of the causative variants in probands

**Pedigree**		**Variant features**							**Population databases**			**Predictive tools**		
		**Gene/RefSeq**	**Chromosome position**	**CDS change**	**Amino acid change**	**Zigosity**	**Location**	**dbSNP**	**1000GP**	**GnomAD**	**Pishgam**	**CADD**	**DANN**	**Mutation Taster**
LC3288		CRB1/ NM_201253.2	Chr1: 197,391,065	c.2107G>T	p.Glu703*	Hom	Exon 6	-	-	-	-	24.9	0.8736	DC
LC1815			Chr1: 197,391,087	c.2128+1G>A	-	Hom	Intron 6	-	-	-	-	26.9	0.9892	DC
LC2708			Chr1: 197,396,730	c.2276_2279dupCTTA	p.Ser758SerfsX33	Hom	Exon 7	-	-	-	-	24.8	-	DC

DC, disease causing

LCA is a genetically and clinically highly heterogeneous disease. Some syndromic features, such as hearing impairment, involvement of nervous system, renal abnormalities, and skeletal anomalies in Senior-Løken syndrome, Joubert syndrome, and conorenal syndrome may accompany with retinal manifestation of LCA. Therefore, these diseases need to be discriminated from LCA as an isolated ocular disease. However, occasionally, this differentiation cannot be achieved by clinical and paraclinical investigations, especially in the early childhood^[^^23^^,^^24^^]^. Moreover, to date, 321 genes and loci on RetNet database have been linked to inherited retinal dystrophies, which some of them such as early onset Retinitis pigmentosa (RP) and CRD may have similar features with LCA. In addition, nearly 30 LCA causative genes have been reported^[^^5^^,^^8^^-^^10^^]^. 

Considering the above mentioned issues, we need a robust, high-throughput, and cost-effective technique for the genetic analysis of LCA or this kind of spectrum disorders. The advent of NGS technology and dramatic decrease in its cost make it an extraordinary diagnostic tool for Mendelian disorders with high locus heterogeneity^[14,15]^ including retinal diseases. In case of genetic diagnosis for retinal diseases, this technology has been successfully applied in panel-based manner or through WES^[^^16^^-^^18^^]^. NGS has higher detection rates than array-based genotyping and is extremely more cost-effective than Sanger- sequencing for LCA disease^[^^25^^]^.

*CRB1* was identified through the candidate gene approach, since its implication has previously been identified in RP12^[^^12^^,^^26^^]^. Indeed, mutations in *CRB1* gene cause a spectrum of hereditary retinal dystrophies, including LCA type 8 (LCA8), early-onset rod-cone dystrophy, RP12, autosomal dominant pigmented paravenous chorioretinal atrophy, recessive RP with para arteriolar preservation, CRD, and isolated autosomal recessive foveal retinoschisis^[^^8^^,^^9^^,^^27^^-^^30^^]^. 

LCA8 (OMIM: # 613835) frequency among LCA cases varies from 0% in Indian to 17% in Spanish patients^[^^1^^,^^28^^]^. Gene-specific indications for *CRB1*associated retinal dystrophy may be some fundus features: deep nummular pigmentations, preservation of the para-arteriolar retinal pigment epithelium, Coats-like vasculopathy, and retinal telangiectasia with exudation^[^^26^^,^^31^^,^^32^^]^. Another paraclinical sign suggesting *CRB1* mutation is retinal thickening on OCT^[^^30^^,^^33^^,^^34^^]^.

The causative gene for LCA8, *CRB1*, is located in 1q31.1 and encodes a 1406-amino acid protein containing 19 epidermal growth factor (EGF)-like domains and three Laminin A globular-like domains in extracellular region, and a short cytoplasmic tail^[^^35^^]^. More than 300 pathogenic variants have been reported in *CRB1* gene, which the most common ones are missense (about 60%)^[^^36^^]^. There are efforts to correlate type of mutation or its location with *CRB1*-related disorders and their severities. Studies attempting to connect specific mutations in *CRB1* to specific phenotypes have not reached remarkable success^[^^27^^,^^31^^]^. However, it seems that null alleles (i.e., nonsense, frameshift deletions/duplication/insertions, and canonical splice site mutations) are mostly common among more severe retinal dystrophies and LCA patients^[^^32^^,^^37^^,^^38^^]^, with the same as our cases. Kuniyoshi *et al.*^[^^39^^]^ have reported three novel null mutations in affected individuals with milder severity, slow progression, and without nystagmus. Another study have shown the combination of two null alleles in 40% of LCA cases, but not in early-onset RP patients^[^^40^^]^. 

To establish phenotype-genotype correlation regarding the location of missense mutations, Beryozkin *et al.*^[^^27^^]^ have studied the phenotype of patients with homozygous missense mutations in *CRB1*. They found that patients with mutations within the Ca-binding EGF-like domains have more severe disease (LCA or early RP) compared to patients with homozygous missense mutations in the laminin AG-like domains. Detected variants in this study are located in EGF-like domain 12 and expected to have severe effects. Several other missense or stop-gained mutations, mostly resulting in LCA, have been reported in this domain^[^^12^^,^^31^^,^^41^^]^.

In summary, there is no straightforward association between type of mutations and their clinical consequences; variability of *CRB1*-related phenotype may correlate with genetic background and modifier loci and even environmental factors rather than type of mutation in *CRB1*^[^^32^^,^^42^^]^. Intrafamilial variability in LCA families has been reported since 1960s^[^^43^^-^^45^^]^. Our proband in pedigree LC3288 did not show nystagmous; it was the first symptom detected in his affected aunt. The same variability has been reported in a Chinese family; one sib was affected by *CRB1*-LCA presented with nystagmous, whereas his affected sister did not^[^^46^^]^. Of 300 reported mutations, 50 were nonsense variants. Besides, 27 nonsenses and 33 small indels, which are located in the downstream of our detected variant and lead to premature termination codons, have previously been reported in HGMD^[^^36^^]^. 

Evidence supports the causative effects of the detected variants: absence in population databases, deleterious effects in computational predictive tools; loss of function as a known mechanism of the disease, co-segregation of the phenotype in three affected members of pedigree LC3288, and specificity of patients’ phenotypes for *CRB1*-related disorders. Therefore, these variants can be classified as pathogenic variants based on American College of Medical Genetics and Genomics guideline for the interpretation of sequence variants^[^^47^^]^.

Consanguineous marriages are prevalent in Iranian population with the rate of 38.6%, which first cousin marriages are the most common form^[^^8^^]^. Therefore, higher prevalence of single gene disorders such as LCA than Western countries could be expected. As a result, the implementation of high-throughput methods such as WES to detect disease-causing variants in diseases with high clinical and genetic heterogeneities in this kind of populations can be important in the early diagnosis of the patients and making informed decision of their relatives in premarriage or preconception genetic counseling.

## References

[1] Chacon-Camacho OF, Zenteno JC (2015). Review and update on the molecular basis of Leber congenital amaurosis. World journal of clinical cases.

[2] Weleber RG, Francis PJ, Trzupek KM, Beattie C, In: Adam MP, Ardinger HH, Pagon RA, Wallace SE, Bean LJH, Stephens K, Amemiya A (2004). Leber Congenital Amaurosis. GeneReviews®.

[3] Fazzi E, Signorini SG, Scelsa B, Bova SM, Lanzi G (2003). Leber's congenital amaurosis: an update. European journal of paediatric neurology.

[4] den Hollander AI, Roepman R, Koenekoop RK, Cremers FP (2008). Leber congenital amaurosis: genes, proteins and disease mechanisms. Progress in retinal and eye research.

[5] Medina CA, Townsend JH, Singh AD (2016). Manual of Retinal Diseases: A Guide to Diagnosis and Management. Switzerland: Springer.

[6] Kumaran N, Moore AT, Weleber RG, Michaelides M (2017). Leber congenital amaurosis/early-onset severe retinal dystrophy: clinical features, molecular genetics and therapeutic interventions. British journal of ophthalmology.

[7] Koenekoop RK (2004). An overview of Leber congenital amaurosis: a model to understand human retinal development. Survey of ophthalmology.

[8] Saadat M, Ansari-Lari M, Farhud D (2004). Short report consanguineous marriage in Iran. Annals of human biology.

[9] Daiger SF, Rossiter B, Greenberg LJ, Christoffels A, Hide W (1998). Data services and software for identifying genes and mutations causing retinal degeneration. Investigative ophthalmology and visual science.

[10] Pavan S, Rommel K, Marquina MEM, Höhn S, Lanneau V, Rath A (2017). Clinical practice guidelines for rare diseases: The orphanet database. PLoS one.

[11] Alves CH, Pellissier LP, Wijnholds J (2014). The CRB1 and adherens junction complex proteins in retinal development and maintenance. Progress in retinal and eye research.

[12] Lotery AJ, Jacobson SG, Fishman GA, Weleber RG, Fulton AB, Namperumalsamy P, Héon E, Levin AV, Grover S, Rosenow JR, Kopp KK, Sheffield VC, Stone EM (2001). Mutations in the CRB1 gene cause Leber congenital amaurosis. Archives of ophthalmology.

[13] Slavotinek AM (2016). The family of crumbs genes and human disease. Molecular syndromology.

[14] Stranneheim H, Wedell A (2016). Exome and genome sequencing: a revolution for the discovery and diagnosis of monogenic disorders. Journal of internal medicine.

[15] Stark Z, Tan TY, Chong B, Brett GR, Yap P, Walsh M, Yeung A, Peters H, Mordaunt D, Cowie S, Amor DJ, Savarirayan R, McGillivray G, Downie L, Ekert PG, Theda C, James PA, Yaplito-Lee J, Ryan MM, Leventer RJ, Creed E, Macciocca I, Bell KM, Oshlack A, Sadedin S, Georgeson P, Anderson C, Thorne N, Melbourne Genomics Health Alliance, Gaff C, White SM (2016). A prospective evaluation of whole-exome sequencing as a first-tier molecular test in infants with suspected monogenic disorders. Genetics in medicine.

[16] Tiwari A, Bahr A, Bähr L, Fleischhauer J, Zinkernagel MS, Winkler N, Barthelmes D, Berger L, Gerth-Kahlert C, Neidhardt J, Berger W (2016). Next generation sequencing based identification of disease-associated mutations in Swiss patients with retinal dystrophies. Scientific reports.

[17] Glöckle N, Kohl S, Mohr J, Scheurenbrand T, Sprecher A, Weisschuh N, Bernd A, Rudolph G, Schubach M, Poloschek C, Zrenner E, Biskup S, Berger W, Wissinger B, Neidhardt J (2014). Panel-based next generation sequencing as a reliable and efficient technique to detect mutations in unselected patients with retinal dystrophies. European journal of human genetics.

[18] Shanks ME, Downes SM, Copley RR, Lise S, Broxholme J, Hudspith KA, Kwasniewska A, Davies WI, Hankins MW, Packham ER, Clouston P, Seller A, Wilkie AO, Taylor JC, Ragoussis J, Németh AH (2013). Next-generation sequencing (NGS) as a diagnostic tool for retinal degeneration reveals a much higher detection rate in early-onset disease. European journal of human genetics.

[19] Wang X, Wang H, Sun V, Tuan HF, Keser V, Wang K, Ren H, Lopez I, Zaneveld JE, Siddiqui S, Bowles S, Khan A, Salvo J, Jacobson SG, Iannaccone A, Wang F, Birch D, Heckenlively JR, Fishman GA, Traboulsi EI, Li Y, Wheaton D, Koenekoop RK, Chen R (2013). Comprehensive molecular diagnosis of 179 Leber congenital amaurosis and juvenile retinitis pigmentosa patients by targeted next generation sequencing. Journal of medical genetics.

[20] Schwarz JM, Cooper DN, Schuelke M, Seelow D (2014). MutationTaster2: mutation prediction for the deep-sequencing age. Nature methods.

[21] Kircher M, Witten DM, Jain P, O'roak BJ, Cooper GM, Shendure J (2014). A general framework for estimating the relative pathogenicity of human genetic variants. Nature genetics.

[22] Quang D, Chen Y, Xie X (2014). DANN: a deep learning approach for annotating the pathogenicity of genetic variants. Bioinformatics.

[23] Fazzi E, Signorini SG, Uggetti C, Bianchi PE, Lanners J, Lanzi G (2005). Towards improved clinical characterization of Leber congenital amaurosis: neurological and systemic findings. American journal of medical genetics Part A.

[24] Casteels I, Spileers W, Demaerel P, Casaer P, De Cock P, Dralands L, Missotten L (1996). Leber congenital amaurosis-differential diagnosis, ophthalmological and neuroradiological report of 18 patients. Neuropediatrics.

[25] Xu Y, Xiao X, Li S, Jia X, Xin W, Wang P, Sun W, Huang L, Guo X, Zhang Q (2016). Molecular genetics of Leber congenital amaurosis in Chinese: new data from 66 probands and mutation overview of 159 probands. Experimental eye research.

[26] den Hollander AI, ten Brink JB, de Kok YJ, van Soest S, van den Born LI, van Driel MA, van de Pol DJ, Payne AM, Bhattacharya SS, Kellner U, Hoyng CB, Westerveld A, Brunner HG, Bleeker-Wagemakers EM, Deutman AF, Heckenlively JR, Cremers FP, Bergen AA (1999). Mutations in a human homologue of Drosophila crumbs cause retinitis pigmentosa (RP12). Nature genetics.

[27] Beryozkin A, Zelinger L, Bandah-Rozenfeld D, Harel A, Strom TA, Merin S, Chowers I, Banin E, Sharon D (2013). Mutations in CRB1 are a relatively common cause of autosomal recessive early-onset retinal degeneration in the Israeli and Palestinian populations. Investigative ophthalmology and visual science.

[28] Ehrenberg M, Pierce EA, Cox GF, Fulton AB (2013). CRB1: one gene, many phenotypes. Seminars ophthalmology.

[29] Shah N, Damani MR, Zhu XS, Bedoukian EC, Bennett J, Maguire AM, Leroy BP (2017). Isolated maculopathy associated with biallelic CRB1 mutations. Ophthalmic genetics.

[30] Talib M, van Schooneveld MJ, van Genderen MM, Wijnholds J, Florijn RJ, Ten Brink JB, Schalij-Delfos NE, Dagnelie G, Cremers FPM, Wolterbeek R, Fiocco M, Thiadens AA, Hoyng CB, Klaver CC, Bergen AA, Boon CJF (2017). Genotypic and phenotypic characteristics of CRB1-associated retinal dystrophies: a long-term follow-up study. Ophthalmology.

[31] Henderson RH, Mackay DS, Li Z, Moradi P, Sergouniotis P, Russell-Eggitt I, Thompson DA, Robson AG, Holder GE, Webster AR, Moore AT (2010). Phenotypic variability in patients with retinal dystrophies due to mutations in CRB1. British journal of ophthalmology.

[32] Bujakowska K, Audo I, Mohand‐Saïd S, Lancelot ME, Antonio A, Germain A, Léveillard T, Letexier M, Saraiva JP, Lonjou C (2012). CRB1 mutations in inherited retinal dystrophies. Human mutation.

[33] Jacobson SG, Cideciyan AV, Aleman TS, Pianta MJ, Sumaroka A, Schwartz SB, Smilko EE, Milam AH, Sheffield VC, Stone EM (2003). Crumbs homolog 1 (CRB1) mutations result in a thick human retina with abnormal lamination. Human molecular genetics.

[34] Kousal B, Dudakova L, Gaillyova R, Hejtmankova M, Diblik P, Michaelides M, Liskova P (2016). Phenotypic features of CRB1-associated early-onset severe retinal dystrophy and the different molecular approaches to identifying the disease-causing variants. Graefes archive for clinical and experimental ophthalmology.

[35] Berman HM, Westbrook J, Feng Z, Gilliland G, Bhat TN, Weissig H, Shindyalov IN, Bourne PE (2000). The Protein Data Bank. Nucleic acids research.

[36] Stenson PD, Ball EV, Mort M, Phillips AD, Shiel JA, Thomas NS, Abeysinghe S, Krawczak M, Cooper DN (2003). Human gene mutation database (HGMD): 2003 update. Human mutation.

[37] Den Hollander AI, Davis J, van der Velde‐Visser SD, Zonneveld MN, Pierrottet CO, Koenekoop RK, Kellner U, Van Den Born LI, Heckenlively JR, Hoyng CB (2004). CRB1 mutation spectrum in inherited retinal dystrophies. Human mutation.

[38] Motta FL, Salles MV, Costa KA, Filippelli-Silva R, Martin RP, Sallum JMF (2017). The correlation between CRB1 variants and the clinical severity of Brazilian patients with different inherited retinal dystrophy phenotypes. Scientific Reports.

[39] Kuniyoshi K, Ikeo K, Sakuramoto H, Furuno M, Yoshitake K, Hatsukawa Y, Nakao A, Tsunoda K, Kusaka S, Shimomura Y (2015). Novel nonsense and splice site mutations in CRB1 gene in two Japanese patients with early-onset retinal dystrophy. Documenta ophthalmologica.

[40] Corton M, Tatu SD, Avila-Fernandez A, Vallespín E, Tapias I, Cantalapiedra D, Blanco-Kelly F, Riveiro-Alvarez R, Bernal S, García-Sandoval B, Baiget M, Ayuso C (2013). High frequency of CRB1 mutations as cause of Early-Onset Retinal Dystrophies in the Spanish population. Orphanet journal of rare diseases.

[41] Li Y, Wang H, Peng J, Gibbs RA, Lewis RA, Lupski JR, Mardon G, Chen R (2009). Mutation survey of known LCA genes and loci in the Saudi Arabian population. Investigative ophthalmology and visual science.

[42] Zernant J, Külm M, Dharmaraj S, den Hollander AI, Perrault I, Preising MN, Lorenz B, Kaplan J, Cremers FP, Maumenee I, Koenekoop RK, Allikmets R (2005). Genotyping microarray (disease chip) for Leber congenital amaurosis: detection of modifier alleles. Investigative ophthalmology and visual science.

[43] Waardenburg P, Schappert‐Kimmijser J (1963). On various recessive biotypes of Leber's congenital amaurosis. Acta ophthalmologica (Copenh).

[44] Tsang SH, Burke T, Oll M, Yzer S, Lee W, Xie YA, Allikmets R (2014). Whole exome sequencing identifies CRB1 defect in an unusual maculopathy phenotype. Ophthalmology.

[45] Jalkh N, Guissart C, Chouery E, Yammine T, El Ali N, Farah HA, Mégarbané A (2014). Report of a novel mutation in CRB1 in a Lebanese family presenting retinal dystrophy. Ophthalmic genetics.

[46] Chen Y, Zhang Q, Shen T, Xiao X, Li S, Guan L, Zhang J, Zhu Z, Yin Y, Wang P, Guo X, Wang J, Zhang Q (2013). Comprehensive mutation analysis by whole-exome sequencing in 41 Chinese families with Leber congenital amaurosis. Investigative ophthalmology and visual science.

[47] Richards S, Aziz N, Bale S, Bick D, Das S, Gastier-Foster J, Grody WW, Hegde M, Lyon E, Spector E, Voelkerding K, Rehm HL, ACMG Laboratory Quality Assurance Committee (2015). Standards and guidelines for the interpretation of sequence variants: a joint consensus recommendation of the American College of Medical Genetics and Genomics and the Association for Molecular Pathology. Genetics in medicine.

